# Overall effectiveness of pneumococcal conjugate vaccines: An economic analysis of PHiD-CV and PCV-13 in the immunization of infants in Italy

**DOI:** 10.1080/21645515.2017.1343773

**Published:** 2017-07-12

**Authors:** Paolo Castiglia, Lorenzo Pradelli, Stefano Castagna, Veronica Freguglia, Giorgio Palù, Susanna Esposito

**Affiliations:** a Department of Biomedical Sciences, University of Sassari, Sassari, Italy; b AdRes, Turin, Italy; c GSK Italy, Verona, Italy; d Department of Molecular Medicine, University of Padova, Padua, Italy; e Pediatric Clinic, University of Perugia, Perugia, Italy

**Keywords:** acute otitis media, community-acquired pneumonia, cost-effectiveness analysis, invasive pneumococcal disease, overall effectiveness, pneumococcal vaccine, PHiD-CV, *Streptococcus pneumoniae*

## Abstract

Pneumococcal diseases are associated with a significant clinical and economic burden. The 7-valent pneumococcal conjugate vaccine (PCV-7) has been used for the immunization of newborns against invasive pneumococcal diseases (IPD) in Italy while now, the pneumococcal non-typeable *Haemophilus influenzae* protein D conjugate vaccine (PHiD-CV) and the 13-valent pneumococcal conjugate vaccine (PCV-13) are available.

The aim of this analysis was to compare the estimated health benefits, cost and cost-effectiveness of immunization strategies vs. non-vaccination in Italy using the concept of overall vaccine effectiveness.

A published Markov model was adapted using local data wherever available to compare the impact of neonatal pneumococcal vaccination on epidemiological and economic burden of invasive and non-invasive pneumococcal diseases, within a cohort of newborns from the Italian National Health Service (NHS) perspective. A 18-year and a 5-year time horizon were considered for the base-case and scenario analysis, respectively.

PHiD-CV and PCV-13 are associated with the most important reduction of the clinical burden, with a potential marginal advantage of PHiD-CV over PCV-13. Compared with no vaccination, PHiD-CV is found on the higher limit of the usually indicated willingness to pay range (30,000 - 50,000€/quality-adjusted life year [QALY] gained), while the incremental cost-effectiveness ratio (ICER) for PCV-13 is slightly above. Compared with PCV-13, PHiD-CV would provide better health outcomes and reduce costs even at parity price, solely due to its differential effect on the incidence of NTHi acute otitis media (AOM). The analysis on a shorter time horizon confirms the direction of the base-case.

## Introduction

Pneumococcal diseases still represent a major public health issue associated with significant clinical and economic burden worldwide. Among young, elderly, and immunocompromised individuals, infection with *Streptococcus pneumoniae* constitutes an important risk factor for several diseases including non-invasive (pneumonia and acute otitis media [AOM]) and invasive pneumococcal diseases (IPD) (mainly meningitis and bacteremia).^1^

Significant changes in pneumococcal disease epidemiology have been observed since the USA^2^ and European countries,^3–5^ including Italy, have introduced pneumococcal conjugate vaccines (PCVs) into national childhood immunization plans.

In Italy, the first PCV (7-valent pneumococcal conjugate vaccine [PCV-7]) was licensed in 2002 and has been included in regional childhood immunization schedules between 2006 and 2010.^6^ Routine childhood immunization program with PCV-7 has significantly influenced the epidemiology of pneumococcal diseases. Despite an observed increase of non-vaccine serotype (NVT) IPD cases, due to serotype replacement, pneumococcal immunization plan resulted in marked incidence reductions of any-serotype IPD, in all age groups.^7–9^ In 2008–2014, in the 0–4 age group, IPD incidence for all serotypes decreased from 7.1 to 2.9/100,000; incidence for vaccine serotypes (VT) decreased from 5.5 to 1.1/100,000, while incidence for NVT increased from 1.6 to 2.0/100,000 (2.5 in 2013). In the >64 age group, IPD incidence increased from 5.3 to 7.5/100,000; VT incidence decreased from 3.9 to 3.2 (4.9 in 2010 and 4.3 in 2013), whereas NVT incidence increased from 1.4 to 4.4/100,000.^10^

To improve immunization against pneumococcal diseases, 2 second-generation higher-valent PCVs (pneumococcal non-typeable *Haemophilus influenzae* protein D conjugate vaccine [PHiD-CV, *Synflorix*, GSK, serotype 1, 4, 5, 6B, 7F, 9V, 14, 18C, 19F, 23F] and 13-valent pneumococcal conjugate vaccine [PCV-13, *Prevenar 13*, Pfizer, serotype 1, 3, 4, 5, 6A, 6B, 7F, 9V, 14, 18C, 19A, 19F, 23F]) were developed. In 2010, a national recommendation by the Italian Ministry of Health replaced PCV-7 with its successor vaccine PCV-13.^11^ Finally, with the aim to achieve and maintain pneumococcal vaccination coverage in newborns ≥ 95%, the National Vaccination Plan 2012–2014 included the active offer of PCV to all newborns at 3, 5–6 and 11–13 months of age.^12^

Currently in Italy, a 23-valent pneumococcal polysaccharide vaccine (PPV23) for the immunization against IPD in adults and children ≥ 2 years is also available.

At the time of initial licensure of the second-generation PCVs, the effectiveness was evaluated on the basis of criteria of non-inferiority to PCV-7 using serological end-points. However, the overall impact of a PCV cannot be predicted solely on the basis of immunological parameters related to the serotypes actually included in the formulation (VT), as there may be differences in the efficacy against each VT, in their level of cross-protection against NVT, in the ability to induce an indirect protection (herd immunity) and in the level of associated serotype replacement.^13^ The persistence of immunity is another variable in estimating impact of vaccination; for PCV, the clinical relevance of immunological response to some PHiD-CV and PCV-13 serotypes is still not clear, and the association between surrogate markers of protection and clinical protection was not always consistent across serotypes and studies.

In fact, in 2012, the WHO position paper stated that the superiority of a vaccine should not be based on the number of serotypes included unless there is evidence that the inclusion of additional serotypes can enhance its effectiveness in specific epidemiological conditions.^1^ Although it may be tempting to consider the clinical impact of a PCV as simply proportional to the number of serotypes in a formulation, the overall impact on invasive disease is in fact a combination of 3 distinct effects: effect on VT disease, effect (if any) on vaccine-related types (VRTs), and degree of NVT disease variation.^14^ The ability of specific PCV to influence each of these 3 effects is a function not only of the VT composition, but also of their conjugation chemistry, the dosage level of each serotype in the conjugate vaccine, and carrier proteins, making predictions difficult.^14^

For the reasons above, in the literature, vaccine effectiveness is increasingly expressed in terms of overall IPD incidence reduction, regardless of the causative serotype, which is the “hard outcome” of high interest to health policymakers.^3^^,^^13^^,^^15–17^

Furthermore, randomized controlled trials (RCTs) and observational studies have confirmed the protection of PCV-7 and PHiD-CV against cross-reactive serotypes, 6A and 19A respectively, and an overall effectiveness of PHiD-CV.^3^^,^^13^^,^^18^ Based on these findings, in 2015–2016, Belgium, Luxembourg and New Zealand have recognized that both PHiD-CV and PCV-13 vaccines are suitable for inclusion on the National Immunization Schedule.^18–20^ In July 2015, the European Medicines Agency's Committee for Medicinal Products for Human Use has expanded the indications of PHiD-CV by including the cross-protection against serotype 19A.^21^ A systematic review of the literature on the impact and effectiveness of PHiD-CV and PCV-13 in Latin America, performed by the Pan American Health Organization (PAHO), has found no evidence of the superiority of one vaccine over the other with regards to impact and effectiveness on hospitalization and mortality reduction in children under 5 years old, considering the outcomes studied, namely death or hospitalizations due to IPD, pneumonia (X-ray-confirmed pneumonia, consolidated, and clinical pneumonia), meningitis or sepsis.^22^

The aim of the present study was to compare the estimated health benefits, cost and cost-effectiveness of childhood immunization strategies with PCV-7, PHiD-CV, and PCV-13 vs. non-vaccination in the Italian context (despite the fact that PCV7 is no longer on the market, it was considered adequate to compare all possible vaccine strategies). Rather than serotype-specific immunization, we have chosen to use overall vaccine effectiveness (OVE) data, as well described by Hausdorff et al. and considered as a new relevant public health parameter.^14^^,^^22^

## Results

### Base-case (18-year time horizon)

Expected total clinical events and health care costs are shown in Table 1.

**Table 1.** Base-case analysis results (18-year time horizon).

	No vaccination	PCV-7	PCV-13	PHiD-CV
Effectiveness (undiscounted)				
Cases of meningitis (n)	117	68	48	48
Cases of bacteremia (n)	207	119	83	83
Cases of pneumonia (n)	318,015	307,149	301,418	301,418
Cases of AOM (n)	1,576,211	1,507,479	1,361,368	1,341,226
Cases of (AOM) sequelae (n)	21	13	9	9
All deaths^*^ (n)	2,608	2,600	2,596	2,596
QALYs	8,092,626	8,093,201	8,094,029	8,094,129
LYs	8,903,903	8,904,035	8,904,086	8,904,086
Costs (undiscounted)				
Vaccine (€)	—	NA	88,977,589	88,977,589
Acute meningitis (€)	943,520	546,103	387,134	387,134
Meningitis sequelae (€)	1,526,213	857,494	590,004	590,004
Bacteremia (€)	658,774	376,648	263,796	263,796
Pneumonia (€)	74,831,401	67,620,401	66,193,669	66,193,669
AOM (€)	73,431,910	69,410,537	60,862,579	59,684,201
Total (€)	151,391,817	NA	217,274,772	216,096,394
Summary discounted outcomes				
QALYs	6,283,557	6,284,049	6,284,780	6,284,870
LYs	6,914,139	6,914,238	6,914,276	6,914,276
Total costs (€)	131,150,394	NA	197,804,453	196,735,985
ICER (€/QALY gained)				
vs. no vaccination	—	—	54,501	49,957
vs. PCV-13	—	—	—	Dominant

* General + disease-specific mortality

AOM: Acute otitis media; ICER: Incremental cost-effectiveness ratio; LYs: Life years; PCV-7: 7-valent pneumococcal conjugate vaccine; PCV-13: 13-valent pneumococcal conjugate vaccine; PHiD-CV: Pneumococcal non-typeable *Haemophilus influenzae* protein D conjugate vaccine; QALYs: Quality-adjusted life years.

The least effective strategy is to not vaccinate, while higher valency vaccines are associated with the most important reduction of the clinical burden, with a potential marginal advantage of PHiD-CV over PCV-13 determined solely when effectiveness in preventing NTHi-caused AOM episodes is taken into account. On the economic side, the least costly strategy is to not vaccinate, while the most expensive is to vaccinate with PCV-13 – nothing can be said for PCV-7, given the absence of a unit cost for the vaccine, no longer available.

ICERs have been calculated for PHiD-CV and PCV-13 vs. no vaccination, and for PHiD-CV vs. PCV-13 (Table 1).

Compared with no vaccination, PHiD-CV is found on the higher limit of the usually indicated willingness to pay (WTP) range (30,000 - 50,000€/QALY gained), while the ICER for PCV-13 is slightly above.

Comparing the 2 higher valency vaccines, PHiD-CV is expected “dominant," according to the pharmacoeconomics jargon, providing better health outcomes and reducing costs even when considering the parity price. As indeed expected from the inputs, the 2 strategies differ only in their effect on AOM. The reduced AOM incidence is expected to be associated with some 90 incremental QALYs and with savings of over one million Euros for an Italian newborn cohort followed over 18 y.

Based on the base-case results, a threshold analysis indicated that PCV-7 would be cost-effective at a WTP of 50,000€/QALY for unit prices up to €8.09. It may be worth indicating that the ICERs vs. PCV-7 at these price levels would be around 57,000 and 50,000€/QALY for PCV-13 and PHiD-CV, respectively.

### Scenario analysis (5-year time horizon)

The analysis on a shorter time horizon confirms the direction of the base-case, and indicates how most of the expected disease burden, in terms of QALY losses and costs, accumulates in the first years. In the incremental analysis of PHiD-CV vs. PCV-13, reduced AOM incidence is expected to be associated with 64 incremental QALYs and with about €900,000 savings for an Italian newborn cohort followed over 5 y (details in Appendix section 1 in Supplemental Material).

### Sensitivity analysis

#### Probabilistic sensitivity analysis (PSA)

**Figure 1.**
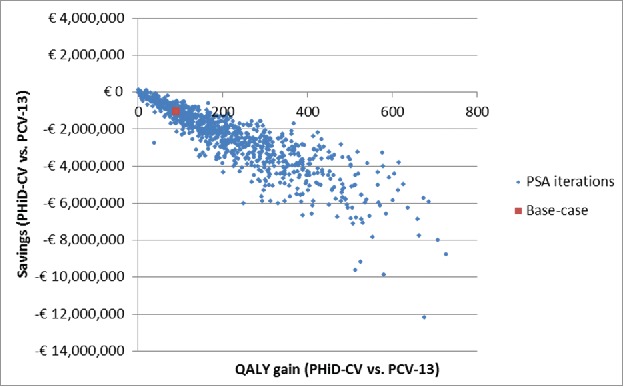
PSA: Scatterplot for PHiD-CV vs. PCV-13 (18-year time horizon). PSA: Probabilistic sensitivity analysis; PCV-13: 13-valent pneumococcal conjugate vaccine; PHiD-CV: Pneumococcal non-typeable *Haemophilus influenzae* protein D conjugate vaccine; QALY: Quality-adjusted life year.

The distribution of expected differences among the active strategies is presented in Fig. 1 as scatterplot of the incremental results of the 1,000 iterations of the PSA. The high correlation among QALY and cost differences is not surprising if one considers that both are related to AOM prevention.

#### Deterministic sensitivity analysis

**Figure 2.**
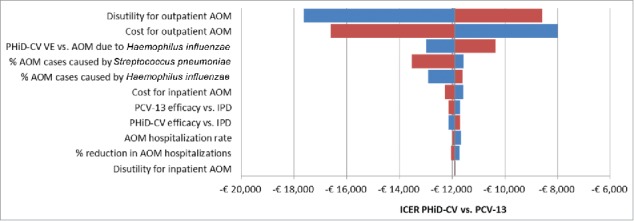
DSA: Tornado diagram of ICER of PHiD-CV vs. PCV-13. AOM: Acute otitis media; DSA: Deterministic sensitivity analysis; ICER: Incremental cost-effectiveness ratio; IPD: Invasive pneumococcal disease; PHiD-CV: Pneumococcal non-typeable *Haemophilus influenzae* protein D conjugate vaccine; PCV-13: 13-valent pneumococcal conjugate vaccine; VE: Vaccine effectiveness.

The tornado diagram depicted in Fig. 2 shows the influence on the output of the model, expressed in terms of ICER of PHiD-CV vs. PCV-13, of variations in input parameters to their extreme values (details in Appendix section – section 1 in Supplemental Material). Parameters are sorted in order of decreasing sensibility of the results to their variations: the most influential parameters, not surprisingly, are those related to the cost and utility of AOM, which is the driver of the expected differences between PHiD-CV and PCV-13. For all values tested, the ICER remains negative, indicating pharmacoeconomic dominance.

### Discussion

Despite the implementation of vaccination programs, pneumococcal diseases continue to be associated with mortality, morbidity and direct medical costs.

Our economic analysis suggests that from the NHS perspective, considering direct costs only in the direct comparison with PCV-13, the vaccination with PHiD-CV is a more slightly cost-effective strategy for infants in Italy, even at parity price. The advantage of PHiD-CV over PCV-13 is determined solely by its superior effectiveness in preventing AOM episodes that is expected to be associated with some 90 incremental QALYs and with savings of over €1 million accruing over 18 y in a single newborns cohort.

The main limitation of our analysis is the absence of direct comparison data between some of the compared strategies; this, however, is inherent with the type of clinical effectiveness studies in public health, and relates to the surrogate parameters sufficient for regulatory purposes. However, we believe to have taken a conservative approach when selecting the input data, and are therefore confident in the direction indicated by our results.

The efficacy of PHiD-CV against AOM has been shown in the COMPAS study^16^ and supported by the study of Prymula et al.^23^ in which a PHiD-CV precursor vaccine (11Pn-PD) was associated with reductions of 33.6%, 51.5%, and 35.6% for all causes AOM, *Streptococcus pneumoniae*-related AOM, and NTHi-related AOM, respectively. Those results have been confirmed by preclinical and clinical studies.^16^^,^^24^ Furthermore, the study of Camilli et al.^25^ reported that vaccination with PCV-7 and PCV-13 have led to a change in the nasopharyngeal flora of children under 6 y of age, increasing the incidence of AOM caused by NTHi. Based on these evidences, although the indication is not present in the European Summary of product characteristics, we decided in this analysis to consider some effectiveness of PHiD-CV against NTHi-related AOM. This is consistent with economic evaluation methodology which recommended to consider all differential aspects of the compared alternatives.^26^ In fact, as indicated by the results of the sensitivity analyses, from a purely economic point of view, the scale of non-invasive pneumococcal disease overwhelms the effects on IPD, for prevention of which universal children vaccination is mainly advocated and implemented. It may be said that the reduction of AOM and pneumonia cases in early childhood is a concomitant health benefit, albeit less clinically crucial, of PCVs, characterized by an impressive payback that allows for the cost-effectiveness of the vaccination programs.

To our knowledge, this is the first study assessing cost-effectiveness of PCVs using OVE as alternative public health parameter, instead of serotype-specific effectiveness, according to the most recent studies which have questioned the evaluation of vaccines based solely on the number of serotypes. Differences in the effectiveness of VT, in the cross-protection, but also in the induction of indirect protection and in the level of replacement may in fact occur.^14^^,^^27^

To date, observational studies of real life impact are available, and confirm the protection of PHiD-CV against cross-reactive serotypes, 19A in particular. This leads to an OVE similar to PCV-13:^3^^,^^13^^,^^28–30^ it has already been said that the protection against IPD is now more a matter of vaccination coverage than of selection of one over the other higher valency PCVs.^14^ Consistently with these findings, some European countries, such as Austria, Finland, Iceland, the Netherlands, Belgium and Sweden (Regions), have considered PHiD-CV equivalent to PCV-13 in terms of IPD protection and therefore adopted vaccination with PHiD-CV.

The use of OVE instead of serotype-specific data has prompted the need for changing some of the methodology of the original simulation model; these changes were mainly related to the overcoming of the need for some assumptions. In particular, there was no further need to assume the size and duration of the indirect protective effect, which was estimated as a balance between cross-protection, herd immunity, and serotype replacement phenomena in earlier, serotype-specific data fed versions of the model. OVE data capture this balance directly. Furthermore, no assumptions on the dynamics of the “ramp-up” of VE were needed anymore, as the OVE data used already represent the mean effectiveness in the 0–4 y population. A possible drawback of these changes, to be taken into consideration when interpreting the results, is that the levels of OVE are applied to a population with potentially different epidemiology than the one in which it was observed.

As for any study, the results presented have to be interpreted in light of their limitations. We already addressed the main ones, namely the absence of direct comparison data, the use of effectiveness data stemming from countries with a possibly different epidemiology than Italy's, and the fact that differences between the 2 high valency vaccines are completely driven by their effect on AOM, which is not their main clinical indication. Furthermore, we underline that the use of a static, as compared with a dynamic, simulation model precludes the possibility to accurately capture the long-term evolution of the epidemiology brought by vaccination programs. However, we took measures to minimize their potential to introduce bias, as described, and are confident in the direction and main conclusion indicated by our results.

### Conclusions

The results of our analysis further corroborate the above mentioned public health choices, indicating that at an equal level of protection against IPD and pneumonia, PHiD-CV may offer additional slight advantages over PCV-13 in terms of AOM prevention. PHiD-CV may allow for slight economic savings, contributing to the sustainability of the vaccination programs from the NHS perspective, even at parity price.

## Methods

A previously published Markov model^31^ was adapted to simulate the impact of neonatal pneumococcal vaccination on epidemiological and economic burden of meningitis, bacteremia, pneumonia, and AOM, within a cohort of newborns in Italy.

Local data were used to populate the model wherever available, and all the assumptions and data sources were based on an in-depth evidence review. The analysis was conducted from the Italian National Health Service (NHS) perspective and compares 2 + 1 regimes of PHiD-CV (*Synflorix*, GSK) and PCV-13 (*Prevenar 13*, Pfizer) (doses administered at 3, 5, and 11 months) vs. PCV-7 (equaled to a strategy able to maintain current epidemiology) and no vaccination (in which a rebound to pre-PCV epidemiology is expected).

Given the uncertainty associated with epidemiological evolution and future available strategies, the usual lifetime horizon was considered to be too long and barely relevant. The time horizon was limited to the whole pediatric age (up to 18 years) for the base-case and to pre-scholar age (up to 5 years, the follow-up time of the clinical studies from which inputs were obtained) as scenario analysis.

### Model structure

**Figure 3.**
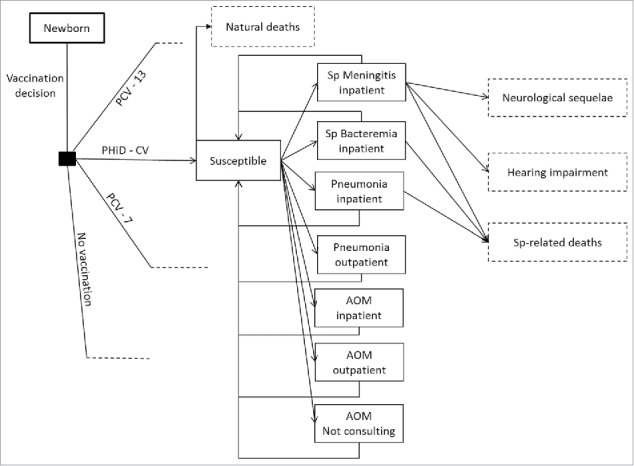
Model flow diagram. Rectangles represent mutually exclusive health states. Dotted rectangles represent absorbing health states and represent the proportion of the population removed from the model. Age-specific incidences are applied monthly to the susceptible population, after accounting for arm-specific VE. Costs and benefits are computed monthly and aggregated over the analyzed time horizon. Non-consulting AOM are accounted for in the quality-of-life impact calculation. No Vaccination: is a counterfactual scenario, in which universal vaccination is not fostered by the health system. It allows assessing the absolute value of PCV vaccination programs, and not only the comparison between 2 specific vaccination products. AOM: Acute otitis media; PCV-7: 7-valent pneumococcal conjugate vaccine; PCV-13: 13-valent pneumococcal conjugate vaccine; PHiD-CV: Pneumococcal non-typeable *Haemophilus influenzae* protein D conjugate vaccine; Sp: *Streptococcus pneumonia.*

The model is a static age-stratified Markov model, considering 12 health states: no disease, meningitis, bacteremia, pneumonia (inpatient and outpatient), AOM (inpatient, outpatient and not-consulting), meningitis-related neurological sequelae and hearing impairment, *Streptococcus pneumoniae*-related and natural deaths (Fig. 3). Individuals of the birth cohort move to health states according to estimated transition probabilities determined by age-specific disease incidence rates (cycle length is one year). Vaccination decreases disease incidence rates and transition probabilities in accordance with vaccine effectiveness for IPD, pneumonia, and AOM. Vaccine effectiveness translates into cost offsets, reduced disutilities, and mortality decline. Costs and quality-adjusted life years (QALYs) specific to each health state were estimated and summarized over the time horizon and converted into a corresponding incremental cost-effectiveness ratio (ICER).

### Population

The population assessed in the model is represented by the cohort of 496,627 newborns in Italy in 2014, as reported by the Italian National Statistics Institute (ISTAT).^32^ Background age-specific mortality rates during the simulation are also taken from ISTAT.^33^

According to the Italian Ministry of Health, PCV vaccine coverage is set at 87.46%.^34^

### Epidemiological data

As detailed in the below *Vaccine effectiveness* section, we use OVE data; therefore incidence of IPD, pneumonia, and AOM epidemiological data used in the model include all serotypes.

The assumption on which epidemiology and vaccine effectiveness inputs are based is that the current epidemiology is a reflection of a quasi-steady-state subsequent to 10 y of pneumococcal conjugate vaccination with PCV-7: the current incidence would be maintained if continuing universal vaccination with PCV-7, it would rebound to pre-PCV-7 levels in case of interruption of the vaccination program, and can be further reduced with vaccines of higher valency, as shown in the clinical studies on which effectiveness inputs are sourced.

#### IPD

To avoid the known underestimation in the nation-wide figures, the age-specific annual incidence of pneumococcal meningitis and bacteremia (referring to 2011) rates were derived from the 7 Italian Regions with active surveillance systems.^10^ In absence of local data, the frequency of meningitis long-term sequelae, and the case fatality rates for meningitis and bacteremia derived from the UK Health Protection Agency^35^ (Table 2).

**Table 2.** Clinical inputs.

Epidemiological data														
	Meningitis			Bacteremia		Hospitalized pneumonia			Non-hospitalized pneumonia		AOM			
Age (years)	Incidence rate (x100,000)^10^	Case fatality ratio (%)^35^	Cases with sequelae (%)^35^	Incidence rate (x100,000)^10^	Case fatality ratio (%)^35^	Hosp. rate (x100,000)^32^^,^^36^	Hosp. rate (%)^32^^,^^36^	Case fatality ratio (%)^36^	GP consult. rate (%)^37^^,^^38^	GP consult. rate (x100,000)^37^^,^^38^	GP consult. rate (x100,000)^39^^,^^40^	Adjustment factor for total AOM cases^39^	Hosp. rate (x100,000)^7^^,^^37^	
<1	4.31	9.2	20.4	4.31*	2.6	550	7.1	0	92.9	7,154	16,482.1	1.2	288.0	
1	1.48	12.9	20.4	3.62	1.7	550	7.1	0	92.9	7,154	22,517.9	1.2	288.0	
2	1.48	17.1	20.4	3.62	2.9	550	7.1	0	92.9	7,154	18,455.4	1.2	133.1	
3	1.48	17.1	20.4	3.62	2.9	550	7.1	0	92.9	7,154	25,767.9	1.2	185.9	
4	1.48	17.1	20.4	3.62	2.9	550	7.1	0	92.9	7,154	19,267.9	1.2	139.0	
5–9	0.60	4.8	20.4	0.90	0	121	7.1	0	92.9	1,570	10,869.2	2.7	78.4	
10–14	0.00	4.8	20.4	0.09	0	121	7.1	0	92.9	1,570	2,470.5	4.1	17.8	
15–18	0.15	11.5	44.4	0.07	0	58	2.6	4	97.4	2,155	740.5	2.3	5.3	
Corrected OVE for post-PCV-7 epidemiology														
			vs. IPD		vs. CAP (hospitalizations)		vs. CAP (GP visits)				vs. any AOM			
PCV-7			0		0		0				0			
PHiD-CV			40		3		3				24			
PCV-13			40		3		3				21			
No vaccination			-100		-26		-4				-10			

* Refers to 1 month-1 y age.

AOM: Acute otitis media; CAP: Community-acquired pneumonia; CFR: Case fatality rate; Consult.: Consultation; GP: General practitioner; Hosp.: Hospitalization; IPD: Invasive pneumococcal disease.

#### Community-acquired pneumonia (CAP)

The overall incidence rate for hospitalized all-cause pneumonia was calculated from age- and gender-specific incidence rates in the Veneto Region during 2004–2012,^36^ weighted for the relative age-group specific proportion of males and females in Italy.^32^ Age-specific case-fatality ratios for hospitalized pneumonia were also taken from Baldo et al.^36^ (Table 2). The incidence rates for non-hospitalized CAP were derived from national general practitioners (GP) and pediatricians databases^37^^,^^38^(Table 2).

#### AOM

The proportion of national AOM cases due to *Streptococcus pneumoniae* (32%) or non-typeable *Haemophilus influenzae* (NTHi – 43%) was taken from the study by Camilli et al.^25^

Incidence of AOM in 0–6-year-olds was elaborated from age-specific incidence rates of AOM in Italy from one local and one multinational study.^39^^,^^40^ In the model, GP consultation rate is corrected with an adjustment factor (1.25) to estimate the true incidence of AOM taking into account the percentage of cases not associated with a visit (Table 2 - details in Appendix section 2 in Supplemental Material).

The hospitalization rates for AOM in 0–2-year-olds were taken from Ansaldi et al.^7^; for older groups it has been calculated from the AOM hospitalization rate reported by the Pedianet 2006 report^37^ (Table 2).

### Vaccine effectiveness

#### Effectiveness against IPD

Based on in depth review of latest evidence showing equivalent protection of PHiD-CV and PCV-13 for IPD and pneumonia, we use OVE data, more accessible than serotype-specific immunization data and a relevant alternative in particular from the Public health perspective for the decision makers.

The Quebec experience was considered as the most appropriate source to extract OVE (75% for PHiD-CV, 65% for PCV-13, 50% for PCV-7) since it includes data for the different vaccines and because these vaccines have been subsequently used in the same healthcare system.^13^ However, in absence of a direct comparison study between PHiD-CV and PCV-13, we deemed a conservative approach to be preferable, and attributed them equal OVE (70%, mean of the 2). This is aligned with other non-comparative studies on PHiD-CV,^3^^,^^16^ and PCV-13,^17^^,^^41^ which report similar OVE (e.g., for PHiD-CV in Jokinen et al. = 80%;^3^ for PCV-13 in Waight et al. = 77% and 74% in 0–2 and 2–4-year-olds, respectively^17^).

Reported OVE was recalculated in consideration of the fact that the assumed 70% OVE refers to the comparison with no vaccination. The effect of PCV-13 and PHiD-CV in PCV-7-pre-exposed populations is less (56%).^17^ Current incidence in Italy reflects about 10 y of universal vaccination: the assumption is that these represent a 50% remaining incidence vs. pre-PCV-7 (50% is the OVE of PCV-7 recorded in Quebec^13^).

Conversely, a “no vaccination” strategy would be expected to result in a return to the pre-PCV epidemiology, with a rebound due to higher circulation of the invasive serotypes now controlled by the herd immunity and ongoing vaccination of newborns. Corrected OVE vs. IPD are presented in Table 2 (details in Appendix section 3 in Supplemental Material).

In accordance with the OVE source used (referring to the entire age group 0–4-year-olds), we attributed a plateau phase of full effectiveness from 0 to 4 y included (the phase is linear), and a declining phase until complete waning at 10 y. This implies that no further reduction in IPD incidence beyond that age is expected when compared with the current epidemiology.

#### Effectiveness against pneumonia

In the absence of comparative vaccine effectiveness estimates on pneumonia, PHiD-CV and PCV-13 are attributed the same reduction in hospitalizations (23%) and in GP visits (7.3%), taken from COMPAS.^16^ This effectiveness level refers to a universal vaccination-naïve population; consistently with the recalculation of vaccine effectiveness against IPD, we adjusted effectiveness taking into account reduced pneumonia incidence following PCV-7^42^ (Table 2 - details in Appendix section 4 in Supplemental Material).

#### Effectiveness against AOM

Vaccine effectiveness against all-cause AOM was calculated by multiplying pathogen-specific vaccine effectiveness by relative prevalence of the causative pathogens as reported in Camilli et al. (32% and 43% of AOM cases are attributable to *Streptococcus pneumoniae* and NTHi, respectively).^25^ For PHiD-CV, the effectiveness against AOM caused by NTHi was taken from COMPAS^16^ while in absence of data, PCV-13 effectiveness against NTHi-caused AOM was assumed equal to PCV-7 effectiveness.^43^

Effectiveness data in *Streptococcus pneumoniae*-caused episodes prevention come from an observational study for PCV-7 and PCV-13,^44^ and from COMPAS for PHiD-CV,^16^ corrected for current epidemiology (Table 2 - details in Appendix section 5 in Supplemental Material).

#### Disutilities

Specific disutilities to each health state were used in the model (Table 3).

**Table 3.** Disutilities used in the model.

Short term	Estimate	Source
Meningitis (in-patient)	0.023	^45^
Bacteremia (in-patient)	0.008	^45^
Bacteremia (out-patient)	0.008	As above
Pneumonia (in-patient)	0.008	As above
Pneumonia (out-patient)	0.006	^45^
AOM (out-patient)	0.005	^46^
AOM/TTP hospitalized	0.005	As above
AOM complications	0.005	As above
Long term	Estimate	Source
Neurological sequelae meningitis	0.400	^47^
Hearing loss from meningitis	0.200	^47^^,^^48^
Meningitis long-term sequelae children	0.269	Calculated in model
Meningitis long-term sequelae adults	0.286	Calculated in model
Bacteremia long-term sequelae children	0.269	As meningitis children
Bacteremia long-term sequelae adults	0.286	As meningitis adults
Hearing loss from AOM	0.090	^49^
AOM long-term sequelae	0.090	As above

AOM: Acute otitis media; TTP: Tympanostomy tube placement.

### Costs

Consistently with the perspective of the Italian NHS, the analysis estimated direct medical costs only (related to vaccine and administration, inpatient/outpatient disease-related treatment and long-term sequelae) (Table 4). All costs were measured in € (2015 values). In the model, the same price of €47.73 per vaccine dose for both PHiD-CV and PCV-13 was applied (maximum price for NHS^50^^,^^51^), because in Italy, the regional tender is based on price only. Health effects and costs were discounted at 3%/year.

**Table 4.** Direct costs estimated in the model.

	Unit cost (€)	Source
PHiD-CV	47.73	Parity price with PCV-13
PCV-13	47.73	Maximum price for NHS^50^^,^^51^
Meningitis - first year (acute episode)	8,067	DRG 560 tariff^52^
Bacteremia - hospitalized	3,176	DRG 417 tariff^52^
Pneumonia - hospitalized	1,948	DRG 91 tariff^52^
AOM hospitalized	662	DRG 70 tariff^52^
Pneumonia - outpatient	97	^53^
AOM GP consultations	76	^53^
Neurological sequelae (per year)	11,249	^35^
Hearing loss (per year)	866	^35^

AOM: Acute otitis media; DRG: Diagnosis-related group; GP: General practitioner; NHS: National Health Service; PCV-13: 13-valent pneumococcal conjugate vaccine; PHiD-CV: pneumococcal non-typeable *Haemophilus influenzae* protein D conjugate vaccine.

## Trademark statement

Synflorix is a trade mark owned by the GSK group of companies. Prevenar is a trade mark of Wyeth LLC.

## Supplementary Material

KHVI_A_1343773_supp.docx

## Abbreviations

AOM: acute otitis media
CAP: community-acquired pneumonia
GP: general practitioner
ICER: incremental cost-effectiveness ratio
IPD: invasive pneumococcal disease
ISTAT: Istituto nazionale di statistica (Italian National Statistics Institute)
NHS: National Health Service
NVT: non-vaccine serotype
NTHi: non-typeable *Haemophilus influenzae*
OVE: overall vaccine effectiveness
PCV: pneumococcal conjugate vaccine
PCV-7: 7-valent pneumococcal conjugate vaccine
PCV-13: 13-valent pneumococcal conjugate vaccine,
PHiD-CV: pneumococcal *Haemophilus influenzae* protein D conjugate vaccine
PPV-23: 23-valent pneumococcal polysaccharide vaccine
PSA: probabilistic sensitivity analysis
QALY: quality-adjusted life years
RCT: randomized controlled trials
VT: vaccine serotype
WHO: World Health Organization
WTP: willingness to pay
