# Supplementary material for: Overall effectiveness of pneumococcal conjugate vaccines: An economic analysis of PHiD-CV and PCV-13 in the immunization of infants in Italy
Source: Hum Vaccin Immunother. 2017 Jul 12;13(10):2307–15. doi: 10.1080/21645515.2017.1343773 (PMC5647981; doi:10.1080/21645515.2017.1343773)
Supplement: KHVI_A_1343773_supp.docx [file khvi-13-10-1343773-s001.docx]

**Overall effectiveness of pneumococcal conjugate vaccines: an economic analysis of PHiD-CV and PCV-13 in the immunization of infants in Italy - Appendix**

# Results

**Table VA. Scenario analysis results (5-year time horizon)**

|  | No vaccination | PCV-7 | PCV-13 | PHiD-CV |
| --- | --- | --- | --- | --- |
| **Effectiveness (undiscounted)** | | | | |
| Cases of meningitis (n) | 95 | 51 | 33 | 33 |
| Cases of bacteremia (n) | 174 | 93 | 61 | 61 |
| Cases of pneumonia (n) | 200,732 | 190,707 | 185,420 | 185,420 |
| Cases of AOM (n) | 637,623 | 590,867 | 491,490 | 477,791 |
| Cases of sequelae (n) | 17 | 9 | 6 | 6 |
| All deaths* (n) | 1,844 | 1,836 | 1,833 | 1,833 |
| QALYs | 2,248,329 | 2,248,656 | 2,249,196 | 2,249,264 |
| LYs | 2,475,574 | 2,475,595 | 2,475,603 | 2,475,603 |
| **Costs (undiscounted)** | | | | |
| Vaccine (€) | - | NA | 88,977,589 | 88,977,589 |
| Acute meningitis (€) | 765,855 | 408,576 | 265,663 | 265,663 |
| Meningitis sequelae (€) | 257,526 | 137,386 | 89,329 | 89,329 |
| Bacteremia (€) | 553,956 | 295,533 | 192,162 | 192,162 |
| Pneumonia (€) | 50,339,820 | 43,687,825 | 42,371,630 | 42,371,630 |
| AOM (€) | 45,206,929 | 41,905,092 | 34,887,184 | 33,919,748 |
| Total (€) | 97,124,085 | NA | 166,783,557 | 165,816,121 |
| **Summary discounted outcomes** | | | | |
| QALYs | 2,092,724 | 2,093,026 | 2,093,525 | 2,093,589 |
| LYs | 2,304,232 | 2,304,251 | 2,304,258 | 2,304,258 |
| Total costs (€) | 90,333,590 | NA | 160,016,199 | 159,121,540 |

* General + disease-specific mortality. QALYs: Quality-adjusted life years; LYs: Life years.

**Table VIA. Deterministic sensitivity analysis – Input parameters**

| **Parameters** | **Base-case** | **Min** | **Max** |
| --- | --- | --- | --- |
| Disutility for inpatient AOM | 0.005 | 0.003 | 0.007 |
| % reduction in AOM hospitalizations | 24% | 19% | 29% |
| AOM hospitalization rate | 1.0 | 0.7 | 1.1 |
| PHiD-CV efficacy vs. IPD | 40% | 32% | 48% |
| PCV-13 efficacy vs. IPD | 40% | 32% | 48% |
| Cost for inpatient AOM | €662 | €428 | €946 |
| % AOM cases caused by *Haemophilus influenzae* | 43% | 38% | 49% |
| % AOM cases caused by *Streptococcus Pneumoniae* | 32% | 25% | 38% |
| PHiD-CV VE vs. AOM due to *Haemophilus influenzae* | 29% | 23% | 35% |
| Cost for outpatient AOM | €76 | €49 | €108 |
| Disutility for outpatient AOM | 0.005 | 0.003 | 0.007 |

# Acute otitis media (AOM) epidemiology

As the primary source, the study by Marchisio et al. ^1^ has been selected, as it reports the data with the needed details up to 6 years of age. This has been integrated with data from the multinational study by Liese et al. in two ways ^2^:

1. The overall AOM incidence in 0-6 years old in Marchisio et al. (calculated as weighted average of the age-specific incidences considering the age structure of the cohort) is 16.8/100 person-years ^1^, while the corresponding figure in Liese et al. (prospective phase) is 19.5 ^2^. An adjustment factor (1.16) has been calculated and applied to the Marchisio-reported figures.
2. For incidence in older age groups, for which no local data were retrieved, the figures have been estimated by applying the rate of Italian AOM over UK AOM in 0-5-year-olds in Liese et al. (195/228= 0.86) to the age-specific AOM incidence figures reported for UK ^3^.

The resulting incidence values for each age group are reported in Table IA.

**Table IA. AOM general practitioners (GP) consultation incidence per age group**

| Age group (years) | GP consultation rate per 100,000 | Source |
| --- | --- | --- |
| <1 | 16,482.1 | Marchisio et al. ^1^* |
| 1 | 22,517.9 | Marchisio et al. ^1^* |
| 2 | 18,455.4 | Marchisio et al. ^1^* |
| 3 | 25,767.9 | Marchisio et al. ^1^* |
| 4 | 19,267.9 | Marchisio et al. ^1^* |
| 5-9 | 10,869.2 | Interpolation** |
| 10-14 | 2,470.5 | Liese et al. ^2^/UK ^3^*** |
| 15-18 | 740.5 | Liese et al. ^2^/UK ^3^*** |

* Adjusted for the underdiagnosis vs. Liese et al.

** Linear interpolation between the 4 years and 10-14 years age groups incidences

*** Relative AOM incidence of Italy vs. UK in Liese et al. applied to UK data

In the model, GP-consultation rate is corrected with an adjustment factor to estimate the true incidence of AOM taking into account the percentage of cases not associated with a visit.

For the age group 0-6 years, the adjustment factor has been directly calculated from the figures reported by Liese et al. ^2^.

Adjustment factor = Total cases/cases with GP visit = (Medically diagnosed cases + parent-reported only cases)/ GP-visited cases = (240+59)/240 = 1.25

For older age groups, for which no local data were retrieved, UK figures have been employed, after adjustment for the relative Italy/UK missed diagnosis incidence derived again from the Liese et al. study ^2^.

Italy adjustment factor = 1.25

UK adjustment factor = 180+145/180= 1.81

Relative adjustment factor = 1.25/1.81 = 0.69

# Effectiveness against IPD

The vaccine effectiveness (VE) has been recalculated according to the formula:

$${{VE}_{PHiD-CV corrected}=1- \frac{{1- VE}_{PHiD-CV naive}}{1- {VE}_{PCV-7}}}$$

Where VE is vaccine effectiveness, and PHiD-CV corrected and naïve refer to the expected (in a PCV-7 exposed population) and observed (in PCV-7 naïve settings) effectiveness levels, respectively. The same formula is used to derive the effectiveness of such a strategy, which is negative.

Recalculated VE are presented in the Table IIA.

**Table IIA. Overall vaccine effectiveness (OVE) vs. invasive pneumococcal disease (IPD)**

|  | **OVE vs. IPD (%)** | | | |
| --- | --- | --- | --- | --- |
|  | PCV-7 | PHiD-CV | PCV-13 | No vacc |
| In PCV-7 naive | 50 ^4^ | 70* ^4^ | 70* ^4^ | 0 |
| Corrected for post-PCV-7 epidemiology | 0 | 40 | 40 | -100 |
| Residual incidence vs. current | 100 | 60 | 60 | 200 |
| Residual incidence vs. no vaccination | 50 | 30 | 30 | 100 |

* Mean of the OVE for PHiD-CV and PCV-13

# Effectiveness against pneumonia

The VE against pneumonia has been recalculated according to the formula:

$${{VE}_{PHiD-CV corrected}=1- \frac{{1- VE}_{PHiD-CV naive}}{1- {VE}_{PCV-7}}}$$

Where VE is vaccine effectiveness, and PHiD-CV corrected and naïve refer to the expected and observed effectiveness levels, respectively.

Recalculated VE are presented in the Table IIIA.

**Table IIIA. OVE vs community-acquired pneumonia (CAP)**

|  | **OVE vs. CAP (%)** | | | |
| --- | --- | --- | --- | --- |
|  | PCV-7 | PHiD-CV | PCV-13 | No vacc |
| **Hospitalizations** | | | | |
| In PCV-7 naive | 21 ^5^ | 23 ^6^ | 23 ^6^ | 0 |
| Corrected for post-PCV-7 epidemiology | 0 | 3 | 3 | -26 |
| Residual incidence vs. current | 100 | 97 | 97 | 126 |
| Residual incidence vs. no vaccination | 80 | 77 | 77 | 100 |
| **GP visits** | | | | |
| In PCV-7 naive | 4 ^5^ | 7.3 ^6^ | 7.3 ^6^ |  |
| Corrected for post-PCV-7 epidemiology | 0 | 3 | 3 | -4 |
| Residual incidence vs. current | 100 | 97 | 97 | 104 |
| Residual incidence vs. no vaccination | 96 | 93 | 93 | 100 |

# Effectiveness against AOM

Aggregate effectiveness against AOM is determined by multiplying pathogen-specific VE by relative prevalence of the causative pathogens ^7^, corrected for the current epidemiology in all comparators (Table IVA).

**Table IVA. OVE vs. AOM**

|  | **OVE vs. AOM (%)** | | | | |
| --- | --- | --- | --- | --- | --- |
|  | PCV-7 | PHiD-CV | PCV-13 | | No vaccination |
| ***Haemophilus* *influenza*e (*Hi*) AOM** | | | | | |
| In PCV-7 naive | -11 ^8^ | 21.5 ^6^ | -11* ^8^ | | 0 |
| Corrected for post-PCV-7 epidemiology | **0** | **29** | **0** | | **10** |
| Residual incidence vs. current | 100 | 71 | 100 | | 90 |
| Residual incidence vs. no vaccination | 111 | 79 | 111 | | 100 |
| ***Streptococcus pneumoniae* (*Sp*) AOM** | | | | | |
| In PCV-7 naive | 31 ^9^ | 56 ^6^ | 77 ^9^ | | 0 |
| Corrected for post-PCV-7 epidemiology | **0** | **36** | **67** | | **-45** |
| Residual incidence vs. current | 100 | 64 | 33 | | 145 |
| Residual incidence vs. no vaccination | 69 | 44 | 23 | | 100 |
| **Any AOM** | | | | | |
| *Sp* species (31.56% of AOM cases ^7^) | 0 | 36 | | 67 | -45 |
| *Hi* (43.18% of AOM cases ^7^) | 0 | 29 | | 0 | 10 |
| **Overall** | **0** | **24** | | **21** | **-10** |

* Assumed equal to PCV-7

**References**

1. Marchisio P, Cantarutti L, Sturkenboom M, Girotto S, Picelli G, Dona D, et al. Burden of acute otitis media in primary care pediatrics in Italy: a secondary data analysis from the Pedianet database. BMC Pediatrics 2012; 12:1-8.

2. Liese J, Silfverdal S, Giaquinto C, CARMONA A, Larcombe J, GARCIA-SICILIA J, et al. Incidence and clinical presentation of acute otitis media in children aged <6 years in European medical practices. Epidemiol Infect 2014; 142:1778-88.

3. Williamson I, Benge S, Mullee M, Little P. Consultations for middle ear disease, antibiotic prescribing and risk factors for reattendance: a case-linked cohort study. Br J Gen Pract 2006; 56:170-5.

4. Deceuninck G, De Serres G, Boulianne N, Lefebvre B, De Wals P. Effectiveness of three pneumococcal conjugate vaccines to prevent invasive pneumococcal disease in Quebec, Canada. Vaccine 2015; 33:2684-9.

5. Black SB, Shinefield HR, Ling S, Hansen J, Fireman B, Spring D, et al. Effectiveness of heptavalent pneumococcal conjugate vaccine in children younger than five years of age for prevention of pneumonia. Pediatr Infect Dis J 2002; 21:810-5.

6. Tregnaghi MW, Sàez-Llorens X, López P, Abate H, Smith E, Pósleman A, et al. Efficacy of Pneumococcal Nontypable *Haemophilus influenzae* Protein D Conjugate Vaccine (PHiD-CV) in Young Latin American Children: A Double-Blind Randomized Controlled Trial. PLoS Med 2014; 11:e1001657.

7. Camilli R, Vescio MF, Giufré M, Daprai L, Garlaschi ML, Cerquetti M, et al. Carriage of Haemophilus influenzae is associated with pneumococcal vaccination in Italian children. Vaccine 2015; 33:4559-64.

8. Eskola J, Kilpi T, Palmu A, Jokinen J, Eerola M, Haapakoski J, et al. Efficacy of a Pneumococcal Conjugate Vaccine against Acute Otitis Media. New England Journal of Medicine: Massachusetts Medical Society, 2001:403-9.

9. Ben-Shimol S, Givon-Lavi N, Leibovitz E, Raiz S, Greenberg D, Dagan R. Near-Elimination of Otitis Media Caused by 13-Valent Pneumococcal Conjugate Vaccine (PCV) Serotypes in Southern Israel Shortly After Sequential Introduction of 7-Valent/13-Valent PCV. Clinical Infectious Diseases 2014; 59:1724-32.
